# Assessment of Generalized Joint Hypermobility and Its Association With Osteoarthritis, BMI, and Age: A Study in Southern Lahore

**DOI:** 10.7759/cureus.55990

**Published:** 2024-03-11

**Authors:** Muhammad Shaheer Qamar, Malik Usman Tahir, Muhammad Shehroz, Anusha Zameer, Amil Islam, Marya Yousaf, Nimra Naseer, Mirza Zeeshan Sikandar

**Affiliations:** 1 Orthopedics, University College of Medicine and Dentistry, Lahore, PAK; 2 Neurology, Central Park Teaching Hospital, Lahore, PAK; 3 Rheumatology, Hameed Latif Teaching Hospital, Lahore, PAK; 4 Physical Medicine and Rehabilitation, Aziz Bhatti Shaheed Teaching Hospital, Gujrat, PAK; 5 Community Medicine, Central Park Medical College, Lahore, PAK; 6 Physical Medicine and Rehabilitation, Central Park Teaching Hospital, Lahore, PAK; 7 Orthopedics and Traumatology, Central Park Medical College, Lahore, PAK

**Keywords:** musculoskeletal health, bmi, generalized joint hypermobility, osteoarthritis, joint hypermobility

## Abstract

Background and objective: Joint hypermobility is a physiological variation in the joint range of motion that allows individuals to move their joints beyond the normal limit. Generalized joint hypermobility (GJH) refers to an increased flexibility observed throughout various joints in the body. In younger individuals, joint hypermobility is often more pronounced, serving as a double-edged sword by providing enhanced flexibility for certain activities while simultaneously increasing the susceptibility to musculoskeletal issues. Weight gain and overactivity of joints (joint hypermobility) are associated with the onset of osteoarthritis (OA), and data for the local populace is lacking. This study aims to assess GJH and OA in young and middle-aged women in southern Lahore.

Methodology: A cross-sectional study recruited 116 diagnosed OA patients through a random convenient sampling method. These patients were assessed for GJH using the Beighton criterion. For the assessment of GJH, the Beighton criterion was used, and for OA, radiographs of knee joints were taken. The Beighton criterion consists of nine movements, and each maneuver is assigned a score of either 0 or 1, resulting in a range from 0 to 9. A chi-square test was used for the group comparison of study variables.

Results: A total of 116 adult females participated, with a mean age of 38.34 ± 9.761 and an age range of 20 to 55 years. GJH was assessed and correlated with age using the chi-square correlation and test. Results indicated that 78 (67.24%) exhibited hypermobility at various joint levels, with a likelihood ratio of 43.336 and a *P*-value of <0.001. GJH and BMI were correlated by employing Pearson chi-square correlation, with Pearson chi-square of 2.51 and *P*-value of 0.112 suggestive of no significant association between BMI and GJH.

Conclusions: The dynamic nature of joint hypermobility emphasizes the need to consider age-related changes when assessing its impact on musculoskeletal health. Assessment and management of hypermobility in patients of OA, especially in females, should be made part of routine practices.

## Introduction

Joint hypermobility is a physiological variation in the joint range of motion that allows individuals to move their joints beyond the normal limit. Generalized joint hypermobility (GJH) refers to an increased flexibility observed throughout various joints in the body [1]. While joint hypermobility can be advantageous in certain activities such as dance or gymnastics, it has been associated with a spectrum of musculoskeletal issues [2].

The prevalence of joint hypermobility varies across populations, with some studies suggesting a higher occurrence in females. Understanding the implications of GJH is crucial, particularly in the context of osteoarthritis (OA), a degenerative joint disease characterized by the breakdown of cartilage, leading to pain, stiffness, and impaired joint function. Studies have shown a link between joint hypermobility and an increased risk of musculoskeletal conditions, including OA [3,4]. However, the influence of factors such as age, body mass index (BMI), and gender on this association remains an area of ongoing investigation.

Age and BMI are two critical parameters that play a pivotal role in unraveling the complexities of joint hypermobility and its potential impact on musculoskeletal health [5]. The prevalence of joint hypermobility may exhibit variations across different age groups, shedding light on the dynamic nature of this physiological variation and pathological changes with aging [6]. In younger individuals, joint hypermobility is often more pronounced, serving as a double-edged sword by providing enhanced flexibility for certain activities while increasing the susceptibility to musculoskeletal issues.

BMI, a measure of body fat based on an individual's weight and height, emerges as another critical parameter in the intricate web of joint hypermobility [7]. Studies suggest a potential correlation between BMI and the manifestation of joint hypermobility, with individuals on the higher end of the BMI spectrum potentially experiencing different musculoskeletal challenges compared to those with lower BMI. This relationship adds layers of complexity to our understanding of joint hypermobility and its consequences as OA [8]. Weight gain and overactivity of joints (joint hypermobility) are associated with the onset of OA and data for the local populace is lacking. Therefore, this study is warranted for the assessment of GJH and OA in young and middle-aged women of southern Lahore.

## Materials and methods

Study overview

Under the STROBE (Strengthening the Reporting of Observational Studies in Epidemiology) guidelines, a cross-sectional study was conducted at Central Park Medical College and Teaching Hospital in Lahore, in collaboration with other teaching hospitals, to assess generalized hypermobility in patients with OA from January 2023 to June 2023 (Appendix). Ethical approval (CPMC/IRB-no/1795) was obtained from the institutional review board of the hospital.

Study population

By using a random convenient sampling method, a total of 116 OA patients were recruited for the study after taking written informed consent (in the native language of patients, that is, Urdu) under the guidelines of the Helsinki Declaration. All the included patients had knee OA as per the Kellgren-Lawrence (K-L) scale and hypermobility (with a Beighton score of 4 or above), while patients of septic arthritis, rheumatoid arthritis, psoriatic arthritis, and neurodegenerative disorders were excluded from the study.

Sample size

A sample size of 116 was calculated using the World Health Organization (WHO) sample size calculator, with a confidence interval of 95%, a margin of error of 5%, and a prevalence of GJH set at 8%.

Study procedure

Patients were provided with an explanation of the study protocols, and sociodemographic details were recorded on a standardized, pre-validated study proforma. The diagnosis of OA was confirmed through radiographs, including anteroposterior views of both knees while standing and lateral views in the supine position. The scoring was conducted based on the K-L scale, performed by a radiologist, and graded from grades 0 to 4 [9].

Study assessment

For the assessment of GJH, the Beighton criterion was employed. This criterion comprises nine movements, and each maneuver is assigned a score of either 0 or 1, resulting in a score ranging from 0 to 9 [10]. The included study maneuvers were as follows: passive dorsiflexion of the right/left fifth finger ≥90°, passive opposition of the right/left thumbs to the forearm, right/left elbow hyperextension ≥10°, passive right and left knee hyperextension ≥10°, and palms on the floor during forward trunk flexion with knees extended. A diagnosis of hypermobility is established with a score of 4 or above.

Statistical analysis

Anonymized data were entered into Microsoft Excel (Microsoft Corporation, Redmond, WA) and checked for errors and duplications. After rechecking and cross-verifying the data, they were imported into Statistical Package Software for Social Sciences, Version 26 (IBM Corp., Armonk, NY). Quantitative data were presented in frequencies and percentages. Age and BMI stratification was done for the assessment of GJH in each group and subsequently in subgroups. The chi-square test was used for group comparisons of movements of the fifth metacarpal joint, knee, and bending over the wrist based on age stratification. Likelihood ratios were computed, and a *P*-value < 0.05 was considered significant. Similarly, Pearson chi-square was used to check the association between groups based on BMI and aforementioned movements, and a *P*-value < 0.05 was set as the cutoff for significance.

## Results

A total of 116 adult females, with a mean age of 38.34 ± 9.761 and an age range of 20 to 55 years, were recruited for the study. Age stratification was performed, resulting in group 1 with an age less than 30 years (*n *= 32, 27.6%), group 2 with an age range of 31 to 40 years (*n *= 46, 39.7%), group 3 with an age range of 41 to 50 years (*n *= 20, 17.2%), and group 4 with an age more than 50 years (*n *= 18, 15.5%). Analysis for a type of particular joint OA was made based on the K-L scale. The results indicated predominant involvement of knee joints in all patients, followed by distal interphalangeal joints (DIPs). Additionally, other joints were also involved, as detailed in Table 1.

**Table 1:** Assessment of particular joint involvement in a study population by employing a chi-square test. GH, glenohumeral

Joint involvement	*n* (%)	*P*-value
Shoulder joint (GH joint)	69 (59.48)	0.003
Knee joint	116 (100)	
Hip joint	46 (39.66)	
Proximal interphalangeal (PIP) joints	12 (10.34)	
Distal interphalangeal (DIP) joints	71 (61.20)	
Lumbar spine	38 (32.8)	

GJH was assessed and correlated with age using the chi-square correlation and test. The results revealed that 78 (67.24%) exhibited hypermobility at various joint levels, with a likelihood ratio of 43.336 and a *P*-value of <0.001. This suggests an association between increasing age and joint hypermobility. Further exploration of the extent of hypermobility in various age groups was conducted, as detailed in Table 2. Passive dorsiflexion of the fifth metacarpophalangeal (MCP) joint was performed and recorded in all four groups, which showed an increase in age is associated with an increase in hypermobility of right MCP with a likelihood ratio of 26.568 and *P*-value of <0.001 (Table 2). Similarly, the same trend was observed in passive dorsiflexion of the left MCP joint with 35.56 and a *P*-value of <0.001, as explained in Table 2. To assess hypermobility at the knee joint level, passive hyperextension of 10° was recorded in both knees, as detailed in Table 2. In group 2, 38 (82.6%) exhibited hyperextension in the right knee and 32 (69.6%) in the left knee. These findings suggest that an increase in age leads to an increase in GJH, particularly at the knee joint level. The likelihood ratio for the right knee was 39.746, with a *P*-value of <0.001, as outlined in Table 2. Similarly, a significant association was noted for the left side, with a *P*-value of <0.001 (Table 2). There was no significant association observed between age groups and the ability to rest the wrist on the floor with straight knees, as explained in Table 2.

**Table 2:** Association between joint hypermobility at various joint levels with age stratification.

Joint movements	Angular positions (degree)	Study group based on age, *n* (%)				Likelihood ratio	*P*-value
		<30 years (*n* = 32)	31-40 years (*n *= 46)	41-50 years (*n *= 20)	>50 years (*n *= 18)		
Passive dorsiflexion of the right fifth metacarpophalangeal joint	<90	0 (0%)	8 (17.4%)	4 (20%)	16 (88.9%)	26.568	
	>90	32 (100%)	38 (82.6%)	16 (80%)	2 (11.1%)		
Passive dorsiflexion of the left fifth metacarpophalangeal joint	<90	0 (0%)	10 (21.7%)	16 (80%)	16 (88.9%)	35.56	
	>90	32 (100%)	36 (78.3%)	4 (20%)	2 (11.1%)		
Hyperextension of the right knee joint	<10	0 (0%)	8 (17.4%)	20 (100%)	10 (55.6%)	39.746	
	>10	32 (100%)	38 (82.6%)	0 (0%)	8 (44.4%)		
Hyperextension of the left knee joint	<10	0 (0%)	14 (30.4%)	18 (90.0%)	14 (77.8%)	33.601	
	>10	32 (100%)	32 (69.6%)	2 (10.0%)	4 (22.2%)		
Resting wrist on the floor with straight knees	Cannot perform	6 (18.8%)	28 (60.9%)	14 (70.0%)	16 (88.9%)	15.055	0.002
	Can perform	26 (81.2%)	18 (39.1%)	6 (30.0%)	2 (11.1%)		

BMI was calculated, and participants were grouped into two categories: group 1 (BMI = 18.5-24.9 kg/m²; *n* = 66) and group 2 (BMI > 25 kg/m²; *n *= 50). The correlation between GJH and BMI was analyzed using the Pearson chi-square correlation, resulting in a Pearson chi-square of 2.51 and a *P*-value of 0.112. These findings suggest no significant association between BMI and GJH. Furthermore, GJH at various joint levels by performing various angular movements was assessed in these groups, as explained in Table 3. It was observed that hyperextension of the right knee was associated with BMI, with a Pearson chi-square of 7.38 and a *P*-value of 0.007. However, no significant associations were found for other variables, as detailed in Table 3.

**Table 3:** Association between joint hypermobility at various joint levels with body mass index.

Joint movements	Angular positions (°)	Study group based on BMI, *n* (%)		Pearson chi-square	*P*-value
		Normal weight (*n *= 66)	Overweight (*n *= 50)		
Passive dorsiflexion of the right fifth metacarpophalangeal joint	<90	16 (24.2%)	12 (24%)	0.001	0.983
	>90	50 (75.8%)	38 (76%)		
Passive dorsiflexion of the left fifth metacarpophalangeal joint	<90	22 (33.3%)	20 (40%)	0.274	0.601
	>90	44 (66.7%)	30 (60%)		
Hyperextension of the right knee joint	<10	12 (18.2%)	24 (48%)	7.385	0.007*
	>10	54 (81.8%)	26 (52%)		
Hyperextension of the left knee joint	<10	20 (30.3%)	26 (52%)	2.798	0.094
	>10	46 (69.70%)	24 (48%)		
Resting Wrist on the floor with straight knees	Cannot perform	36 (54.5%)	30 (60%)	0.414	0.520
	Can perform	30 (45.5%)	20 (40%)		

## Discussion

Our investigation into the relationship between GJH, OA, BMI, and age in young and middle-aged women has provided valuable insights into a complex interplay of factors influencing musculoskeletal health. Our study aligns with previous research, suggesting a higher prevalence of joint hypermobility in females. This consistent observation across studies emphasizes the gender-specific nature of joint hypermobility, indicating potential biological and hormonal influences. The prevalence rate of 67.24% in our study is comparable to the rates reported in studies by Remvig et al. [11] and Castori et al. [12], underscoring the widespread nature of joint hypermobility in diverse populations.

Our findings regarding the association between age and joint hypermobility corroborate existing literature, highlighting the dynamic nature of this physiological variation. The trend of increased hypermobility with age, particularly at the knee joint, resonates with studies conducted by Juul-Kristensen et al. [13] and Grahame et al. [14]. These studies, like ours, emphasize the importance of considering age-related changes when assessing joint hypermobility. The heightened flexibility in younger individuals, while advantageous for certain activities, may evolve with age, influencing the susceptibility to musculoskeletal issues like knee OA.

Our study, although not demonstrating an overall significant association between BMI and joint hypermobility, identified a noteworthy correlation between higher BMI and hyperextension of the right knee joint. This specific association adds a novel dimension to our understanding of the impact of body composition on joint flexibility. Interestingly, our findings align with the work of Fatoye et al. [15], who reported a significant association between BMI and joint hypermobility in their study on adult Nigerians. The consistency in these findings across different populations underscores the relevance of considering BMI as a potential influencing factor in joint hypermobility.

Our study delved into joint-specific manifestations of hypermobility, focusing on the fifth MCP joint and knee joint. The associations identified in our research mirror those reported by Russek [16], who explored joint hypermobility in dancers. Both studies underscore the importance of evaluating hypermobility in a site-specific manner, recognizing that different joints may exhibit distinct patterns of flexibility. The observed gender disparities in joint hypermobility prevalence align with studies by Hakim et al. [17] and Remvig et al. [11], emphasizing the need for gender-specific considerations in musculoskeletal research. While our study focused on young and middle-aged women, these findings resonate with broader discussions on musculoskeletal health, acknowledging the varied impact of joint hypermobility across different demographic groups.

While our study provides valuable insights, it is not without limitations. The cross-sectional design limits our ability to establish causation, and longitudinal studies are warranted to unravel the temporal relationships between joint hypermobility, OA, BMI, and age. Additionally, the study's focus on young and middle-aged women may restrict the generalizability of findings to other demographic groups. Future research should strive for more comprehensive inclusion criteria to capture the diverse manifestations of joint hypermobility across populations.

## Conclusions

The dynamic nature of joint hypermobility emphasizes the need to consider age-related changes when assessing its impact on musculoskeletal health. The nuanced correlation between a higher BMI and specific joint manifestations, particularly in knee joints, was observed. The assessment and management of hypermobility in patients with osteoarthritis, especially in females, should be integrated into routine practices. Moving forward, longitudinal studies encompassing diverse demographic groups are warranted to unravel the complex interplay of factors influencing musculoskeletal health.

## Appendices

Appendix

**Table 4:** STROBE guidelines. STROBE, Strengthening the Reporting of Observational Studies in Epidemiology

	Item No.	Recommendation	Page No.	Relevant text from the manuscript
Title and abstract	1	(a) Indicate the study’s design with a commonly used term in the title or the abstract	Page 1	A cross-sectional assessment of Generalized joint hypermobility and osteoarthritis in young and middle-aged women of Southern Lahore.
		(b) Provide in the abstract an informative and balanced summary of what was done and what was found	Page 2	
Introduction				
Background/rationale	2	Explain the scientific background and rationale for the investigation being reported	Page 3	Weight gain and overactivity of joints (joint hypermobility) are associated with onset of osteoarthritis
Objectives	3	State specific objectives, including any prespecified hypotheses	Page 3	for the assessment of generalized joint hypermobility and osteoarthritis in young and middle-aged women of southern Lahore.
Methods				
Study design	4	Present key elements of study design early in the paper	Page 4	Under the STROBE guidelines, a cross-sectional study was conducted
Setting	5	Describe the setting, locations, and relevant dates, including periods of recruitment, exposure, follow-up, and data collection	Page 4	the Central Park Medical College & Teaching Lahore in collaboration with other teaching hospitals for the assessment of generalized hypermobility in the patients of osteoarthritis from January 2023 to June 2023.
Participants	6	(a) Cohort study—Give the eligibility criteria, and the sources and methods of selection of participants. Describe methods of follow-up Case-control study—Give the eligibility criteria, and the sources and methods of case ascertainment and control selection. Give the rationale for the choice of cases and controls Cross-sectional study—Give the eligibility criteria, and the sources and methods of selection of participants	Page 4	A total of 116 osteoarthritis patients were recruited for the study after taking written informed consent (in native language of patients: Urdu language) under the guidelines of Helsinki Declaration. All the included patients were having knee OA as per Kellgren-Lawrence (K-L) scale and hypermobility (with beighton score 4 or above)
		(b) Cohort study—For matched studies, give matching criteria and number of exposed and unexposed Case-control study—For matched studies, give matching criteria and the number of controls per case		
Variables	7	Clearly define all outcomes, exposures, predictors, potential confounders, and effect modifiers. Give diagnostic criteria, if applicable	Page 4	Age, BMI, Osteoarthritis and joint hypermobility.
Data sources/ measurement	8*	For each variable of interest, give sources of data and details of methods of assessment (measurement). Describe comparability of assessment methods if there is more than one group	Page 4	Kellgren-Lawrence (K-L) scale beighton scoring
Bias	9	Describe any efforts to address potential sources of bias		
Study size	10	Explain how the study size was arrived at	Page 4	Sample size of 116 was calculated by using WHO sample size calculator with confidence interval of 95% margin of error at 5% and prevalence of GJH of 8 percent.

Quantitative variables	11	Explain how quantitative variables were handled in the analyses. If applicable, describe which groupings were chosen and why	Page 4	Chi-Square test was employed
Statistical methods	12	(a) Describe all statistical methods, including those used to control for confounding	Page 4 and 5	Chi-Square test and correlation was employed
		(b) Describe any methods used to examine subgroups and interactions		
		(c) Explain how missing data were addressed		
		(d) Cohort study—If applicable, explain how loss to follow-up was addressed Case-control study—If applicable, explain how matching of cases and controls was addressed Cross-sectional study—If applicable, describe analytical methods taking account of sampling strategy		
		(e) Describe any sensitivity analyses		
Results				
Participants	13*	(a) Report numbers of individuals at each stage of study—eg numbers potentially eligible, examined for eligibility, confirmed eligible, included in the study, completing follow-up, and analysed	Page 6	A total of 116 adult females
		(b) Give reasons for non-participation at each stage		
		(c) Consider use of a flow diagram		
Descriptive data	14*	(a) Give characteristics of study participants (eg demographic, clinical, social) and information on exposures and potential confounders	Page 6	particular joint osteoarthritis
		(b) Indicate number of participants with missing data for each variable of interest		
		(c) Cohort study—Summarise follow-up time (eg, average and total amount)		
Outcome data	15*	Cohort study—Report numbers of outcome events or summary measures over time		
		Case-control study—Report numbers in each exposure category, or summary measures of exposure		
		Cross-sectional study—Report numbers of outcome events or summary measures	Page 6	in which 67.24% (n=78) showed hypermobility
Main results	16	(a) Give unadjusted estimates and, if applicable, confounder-adjusted estimates and their precision (eg, 95% confidence interval). Make clear which confounders were adjusted for and why they were included	Page 7	in which 67.24% (n=78) showed hypermobility
		(b) Report category boundaries when continuous variables were categorized		
		(c) If relevant, consider translating estimates of relative risk into absolute risk for a meaningful time period		

Other analyses	17	Report other analyses done—eg analyses of subgroups and interactions, and sensitivity analyses		
Discussion				
Key results	18	Summarise key results with reference to study objectives	Page 8	the gender-specific nature of joint hypermobility, indicating potential biological and hormonal influences. The prevalence rate of 67.24%
Limitations	19	Discuss limitations of the study, taking into account sources of potential bias or imprecision. Discuss both direction and magnitude of any potential bias	Page 8,9	The cross-sectional design limits our ability to establish causation, and longitudinal studies are warranted to unravel the temporal relationships between joint hypermobility, osteoarthritis, BMI, and age. Additionally, the study's focus on young and middle-aged women may restrict the generalizability of findings to other demographic groups.
Interpretation	20	Give a cautious overall interpretation of results considering objectives, limitations, multiplicity of analyses, results from similar studies, and other relevant evidence	Page 9	The cross-sectional design limits our ability to establish causation, and longitudinal studies are warranted to unravel the temporal relationships between joint hypermobility, osteoarthritis, BMI, and age. Additionally, the study's focus on young and middle-aged women may restrict the generalizability of findings to other demographic groups.
Generalisability	21	Discuss the generalisability (external validity) of the study results	Page 9	
Other information				
Funding	22	Give the source of funding and the role of the funders for the present study and, if applicable, for the original study on which the present article is based	Page 9	
